# Urothelial Carcinoma in a Cat with Bilateral Ectopic Ureters

**DOI:** 10.3390/ani16182950

**Published:** 2026-09-19

**Authors:** Camilla Piazza, Emily Seidel, Marc Dhumeaux, Rosario Vallefuoco, Caroline Fina, Hayley Ronaldson, Elizabeth Alloway, Charlotte Dye

**Affiliations:** 1 Blaise Referrals, Birmingham B45 9UA, UK; 2 Pride Veterinary Referrals, Derby DE24 8HX, UK; 3 Internal Medicine, School of Veterinary Medicine, Royal Veterinary College, Hatfield AL9 7TA, UK; 4 VPG (Veterinary Pathology Group) Histology, Bristol BS16 1EJ, UK

**Keywords:** urothelial carcinoma, ectopic ureter, ureteral obstruction, carcinomatosis

## Abstract

A 10-year-old neutered female Maine Coon was presented with haematuria, weight loss, polyuria/polydipsia and progressive azotaemia. Diagnostic imaging revealed bilateral renal pelvic dilation, ureteral distension and ectopic ureters. Bacterial pyelonephritis was diagnosed, but the azotaemia progressed despite antibiotic therapy, and suspected ureteral obstruction prompted surgical ureteral reimplantation. The visualisation of omental nodules and thickening of the ectopic right ureterovesicular junction intraoperatively raised suspicion of malignancy, and histopathology revealed urothelial carcinoma with carcinomatosis. The cat was euthanised postoperatively due to clinical deterioration, characterised by persistent oliguria and progressive azotemia. Clinicians should consider malignancy as a differential in cats with ectopic ureters and an atypical or refractory clinical course.

## Simple Summary

Ectopic ureters represent congenital malformations in which one or both ureters terminate outside the bladder trigone, most often within the urethra or vagina, and are a well-recognised cause of urinary incontinence, ascending infection, and hydronephrosis in young dogs and cats. In humans, chronic urinary stasis secondary to congenital urinary tract abnormalities has been proposed as a potential factor contributing to the development of urothelial carcinoma; however, the co-existence of ectopic ureters and urinary tract malignancy has not previously been reported in veterinary patients. This report describes a case of urothelial carcinoma with peritoneal carcinomatosis arising in a cat with bilateral ectopic ureters. Clinicians should consider malignancy as a differential in cats with ectopic ureters and an atypical or refractory clinical course.

## 1. Introduction

Urothelial carcinoma (UC) is a neoplasm arising from the mucosal surface of the urinary bladder, urethra, ureters, and the renal tubules, calyces and pelvis. It is commonly documented in humans and dogs, most often arising from the lateral bladder wall and bladder trigone respectively [1].

In contrast, it has a low reported incidence in cats, with the larger case series suggesting a more variable site of origin [2,3,4,5]; the remaining literature is restricted to single case reports. Whilst risk factors for UC in cats remain poorly documented, several factors have been identified in other species. In dogs, these include exposure to insecticides, obesity, cyclophosphamide administration, female sex, and breed predisposition [6,7]. In humans, risk factors include tobacco and alcohol use, chemical exposure, chemotherapy and radiotherapy, male sex, advanced age and genetics [8]. Importantly, chronic bladder inflammation, urinary stasis, urolithiasis and congenital abnormalities of the urinary tract have been proposed as specific contributors to urinary tract neoplasia in humans [9,10].

Ectopic ureters are a congenital anomaly in which one or both ureters terminate distal to the normal insertion at the bladder trigone, often resulting in urinary incontinence [11]. Whilst considered responsible for up to 23% of urinary incontinence in dogs [12], ectopic ureters are rarely reported in cats. It is unclear whether this reflects a true species difference, or whether it represents underdiagnosis and under-reporting in cats. In one retrospective case series, ureteral ectopia was identified in 10 of 19 cats presenting with congenital urinary incontinence [13], while a separate study reported no evidence of ureteral ectopia in a cohort of 35 cats with incontinence [14]. In addition to urinary incontinence, ectopic ureters predispose to urinary stasis and vesicoureteral reflux, leading to an increased risk of infection and urolithiasis [15]. Consequent chronic inflammation can result cellular metaplasia which, in humans, is proposed as a predisposing factor for neoplastic transformation [16]. Indeed, there are multiple reports in the human literature of UC occurring in association with ectopic ureters [17,18,19,20,21,22].

In contrast, no association has been ascertained in veterinary species, with only a single case report describing a dog with concurrent ureteral UC and an orthotopic ureterocele [23]. This report describes the first documented incidence of coexistent ectopic ureters and UC in a cat.

## 2. Case Description

A 10-year-old, 7.2 kg, spayed female Maine Coon was referred to a veterinary hospital in the United Kingdom with a one-month history of haematuria, stranguria, pollakiuria, weight loss, polyuria, polydipsia, and progressive azotaemia. The patient had a five-year history of mild azotaemia and isosthenuria, previously attributed to chronic kidney disease. At the time of presentation, she was fed a renal diet exclusively and was receiving benazepril (0.7 mg/kg PO q24h). Two weeks prior to presentation, the patient had completed a 14-day course of cephalexin (15 mg/kg PO q12h) following a positive voided urine culture. Although the cat’s appetite had improved, ongoing haematuria and worsening azotaemia prompted subsequent referral.

On presentation, the cat was bright, alert and responsive, with a body condition score of 5/9 and mild generalised muscle atrophy. Cardiorespiratory auscultation was unremarkable. The patient’s temperament precluded comprehensive conscious examination, but further evaluation under anaesthesia was unremarkable. In particular, examination of the external genitalia revealed no abnormalities. A complete blood count identified mild normocytic, normochromic, non-regenerative anaemia (haematocrit 22.2% [RI 30.3–42.3]), serum biochemistry revealed mild azotaemia (creatinine 272 µmol/L [RI 0–150]; urea 20.6 mmol/L [RI 2.70–9.2]), and electrolytes were within normal limits. Mild ionised hypocalcaemia was also documented (1.12 mmol/L, RI 1.20–1.32). Blood pressure measured using an oscillometric device according to the ISFM Consensus Guidelines [24] indicated a prehypertensive state [25]: average systolic 153 mmHg, diastolic 97 mmHg, mean 111 mmHg. No evidence of hypertensive retinopathy was identified on ophthalmic examination.

**Figure 1:**
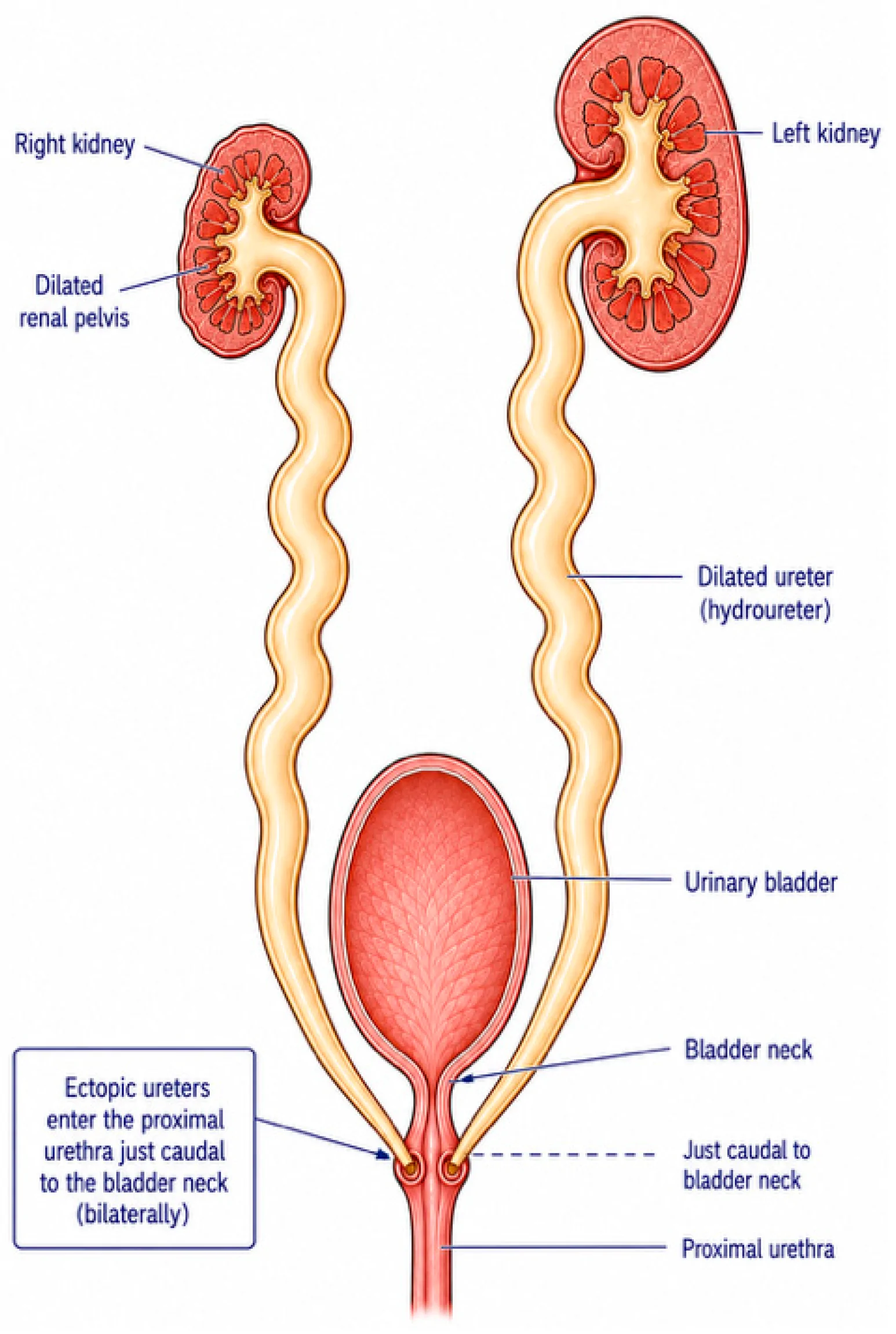
Schematic illustration of abnormal urinary tract anatomy in 10-year-old Maine Coon with ectopic ureters.

Abdominal ultrasound performed under sedation by a board-certified radiologist (GE Logic S7, Seongnam, Republic of Korea) revealed bilateral asymmetrical kidneys with reduced corticomedullary distinction. Both renal pelvises were moderately distended (up to 0.8 cm), and a small nephrolith (0.2 × 0.3 cm) was identified within the right renal pelvis. The left kidney size was otherwise unremarkable, being considered normal considering the breed and body weight (length 5.0 cm) [26]. The right kidney was significantly smaller (length 3.7 cm) with irregular margins. The perirenal fat was moderately thickened and hyperechoic, with minimal associated retroperitoneal effusion. Both ureters were diffusely and moderately distended (up to 0.4 cm in diameter) and displayed ectopic positioning, coursing caudally beyond the bladder neck and entering the proximal urethra just distal to the trigone. No intramural segment was evident, and no ureteral jets were identified within the bladder. The urinary bladder was moderately distended with anechoic urine, and the bladder wall was unremarkable in thickness and architecture. A schematic illustration of the urinary tract abnormalities is shown in Figure 1.

Since the bilateral pelvic dilation and retroperitoneal changes raised suspicion of pyelonephritis, cystocentesis and bilateral pyelocentesis were performed. The patient was positioned in dorsal recumbency, and urine was aspirated from the urinary bladder using a 22-gauge needle under ultrasound guidance. A 22-gauge needle was then percutaneously advanced into each renal pelvis under ultrasound guidance via a paralumbar approach. Urine was aspirated following sonographic confirmation of correct needle tip positioning, with no associated complications. Urinalysis from all three sites indicated marked haematuria and pyuria; the sample from the right renal pelvis exhibited the most pronounced abnormalities, including marked proteinuria, alkaline pH, and active sediment. Culture of the urine from the bladder and from the left renal pelvis indicated growth of *E. coli* (Table 1). The patient was hospitalised for 24 h and received intravenous marbofloxacin (2 mg/kg SID), selected as empirical therapy pending the urine culture and susceptibility results, in accordance with ISCAID guidelines [27]. Benazepril was discontinued. Oral marbofloxacin (2 mg/kg SID) was continued at the time of discharge, supported by the antibiogram results. At the one-week follow-up, the owner reported marked improvement in demeanour and appetite, with complete resolution of the lower urinary tract signs. Antibiotic therapy was continued for an additional three weeks, until completion of a four-week course.

At the four-week re-evaluation, whilst the cat was still receiving marbofloxacin, the owner reported no clinical concerns, and physical examination was unremarkable. However, haematology showed progression of the normocytic, normochromic, non-regenerative anaemia (HCT 17.8% [RI 30.3–42.3]), and serum biochemistry revealed significant worsening of the azotaemia (creatinine 494 µmol/L [RI 0–150]; urea 36.2 mmol/L [RI 2.70–9.2]). Repeat abdominal ultrasonography revealed progression of the urinary tract abnormalities. The left renal pelvic dilation had increased to 2.1 cm, with worsened proximal ureteral dilation (0.35–0.5 cm) and anechoic content as well as mild wall thickening. The right kidney remained small (3.7 cm) and misshapen, with greater pelvic dilation (1.3 cm), medullary hyperechogenicity, and corticomedullary atrophy of the cranial pole. The right ureter was dilated (5 mm) with mild wall thickening. Ectopic termination of both ureters at the level of the proximal urethra was visualised as previously. Marked right-sided retroperitoneal changes persisted, with severe hyperechogenicity and heterogeneous fat. Urine samples were collected via ultrasound-guided cystocentesis and bilateral pyelocentesis as previously described, with all samples showing an active sediment but negative cultures.

Further worsening of the azotaemia was documented the following day (creatinine 916 µmol/L [RI 0–150]; urea 61.23 mmol/L [RI 2.7–9.2]), and, given the progressive renal pelvic dilation, ultrasound-guided antegrade pyelography was performed under anaesthesia to search for potential urinary obstruction. The patient was positioned in dorsal recumbency, and 22 g needles were percutaneously directed into the renal pelvises bilaterally via a paralumbar approach. Maintaining ultrasound guidance using a convex array probe, 5 ml of iodinated contrast medium (Iohexol 300 mg I/mL Omnipaque^®^, GE Healthcare, Chicago, IL, USA) was instilled into each renal pelvis, without complications. Orthogonal abdominal radiographs taken at the time of injection, and at 5, 10, and 15 min post-injection, confirmed bilateral pelvic dilation and identified sequential contrast filling throughout persistently dilated ureters with an irregular, slightly saccular contour. The proximal and caudal segments of the right ureter showed markedly irregular, ill-defined margins, while the remaining ureteral portions were well-delineated. Persistent bilateral narrowing of the terminal ureteral segments and an absence of contrast within the urinary bladder and urethra throughout the 15 min imaging window despite filling of the distal ureters was suggestive of urinary obstruction at the level of the vesicoureteral junctions. However, delayed urinary excretion associated with renal compromise could not be completely excluded.

**Figure 2:**
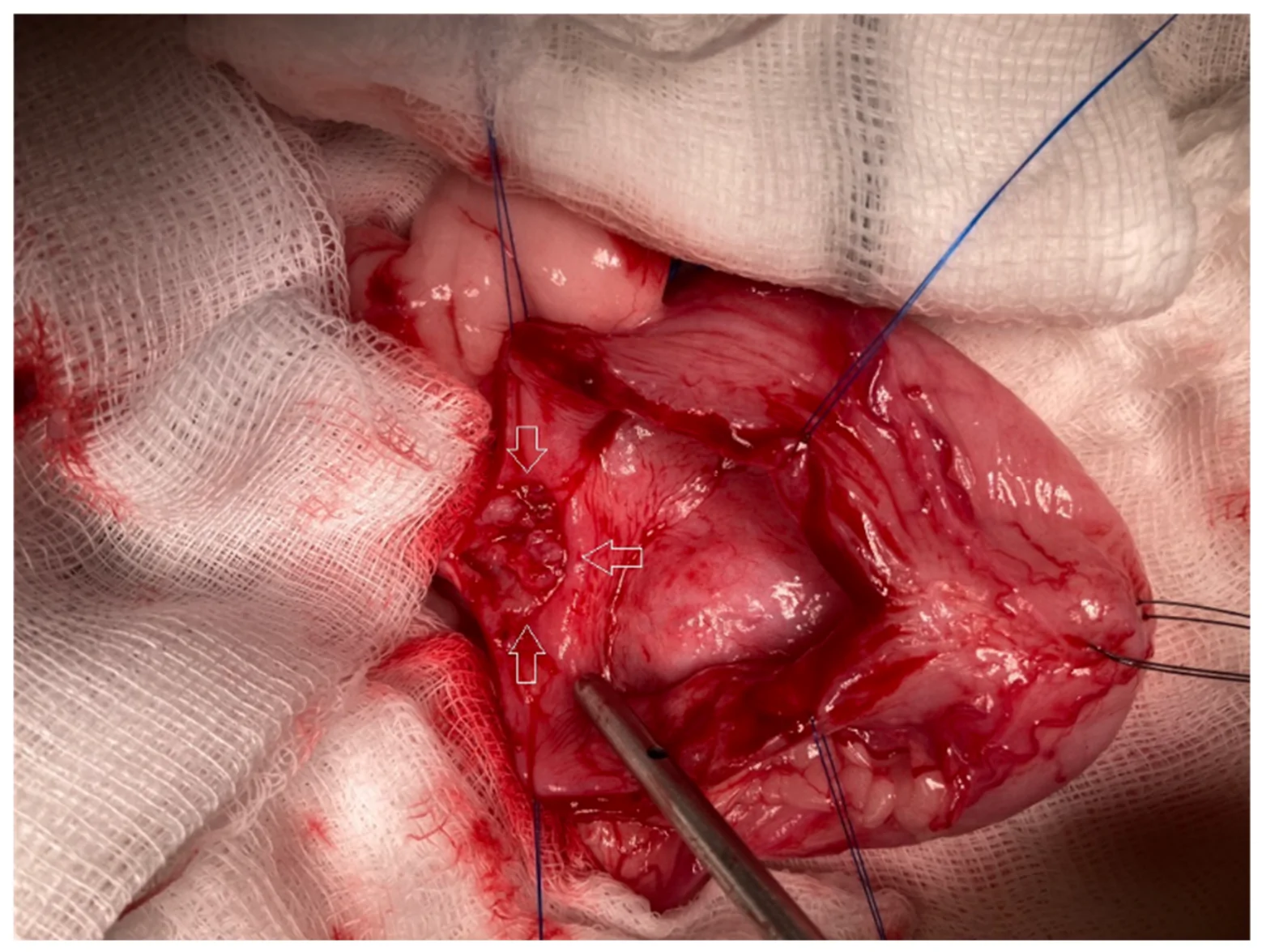
Intraoperative view of proliferative lesion at ureterovesical junction in 10-year-old Maine Coon with ectopic ureters and urothelial carcinoma. White arrows indicate the right ureterovesical junction, which had a proliferative appearance.

**Figure 3:**
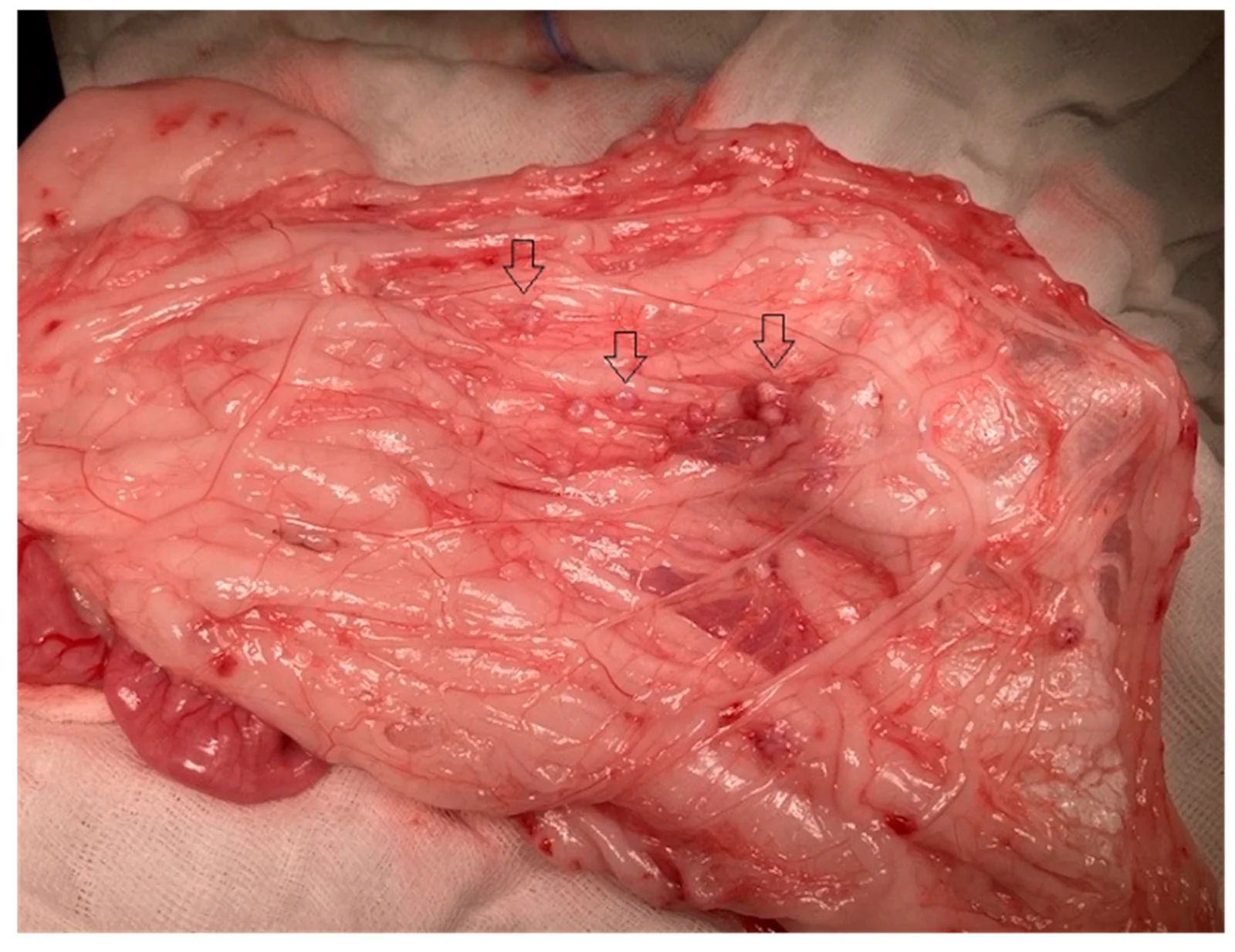
Intraoperative appearance of pale, ill-defined, omental nodules in 10-year-old Maine Coon with ectopic ureters and urothelial carcinoma.

Based on the suspicion of bilateral ureteral obstruction, a midline coeliotomy was performed for urinary tract exploration. A ventral cystotomy extending to the proximal urethra was performed to identify and visualise the ectopic ureterovesical junctions, both of which were located distal to the expected anatomical position, entering at the level of the proximal urethra with no intramural segment. Both ureters were markedly dilated, with the distal right ureter being palpably thickened. A palpable firm thickening was also present at the ectopic entry point of the ureters into the proximal urethra, raising concern of an underlying infiltrative lesion versus chronic inflammatory change (Figure 2). The bladder wall was diffusely thickened. The right kidney was grossly distended, there was a small amount of ipsilateral perirenal fluid, and the right retroperitoneal space was oedematous. In additional to the urinary tract pathology, small pale nodules were scattered throughout the omentum (Figure 3).

Both ureters were ligated and transected as distally as possible, close to their ectopic insertion. An immediate flow of urine was observed from the left ureter following transection, but minimal urine production was visualised from the right ureter. A tomcat catheter was passed in a retrograde fashion into each renal pelvis to allow flushing with saline and check patency of the ureters. Bilateral ureteroneocystostomy was then performed to relocate the ureteral openings to an anatomically appropriate site: both ureters were reimplanted into the dorsal aspect of the bladder near the apex, with the ureteral ends spatulated and anastomosed to the bladder mucosa using 4-0 poliglecaprone (Monocryl, Ethicon, Somerville, NJ, USA) in a simple interrupted pattern. No peristaltic ureteral activity was observed throughout the procedure. Reimplantation was considered technically successful on the left side based on visible urine flow through the anastomosis. Minimal flow from the right ureter was attributed to the severity of pre-existing renal pathology rather than a technical failure of the reimplantation, but, although patent whilst flushing, the function of the right ureteroneocystostomy could not be fully confirmed intraoperatively. Biopsies of the omental nodules and bladder wall were submitted for histopathology along with the abnormal right ureteral tissue harvested from the ectopic junction. The cystotomy was closed with 3-0 Monocryl in a simple continuous pattern, and the abdomen was lavaged with warm saline prior to routine three-layer closure with intradermal skin sutures. A urinary catheter and oesophagostomy tube were placed prior to anaesthetic recovery for ongoing monitoring of urine output and nutritional support respectively.

Anaesthetic recovery was uneventful, and the cat received intravenous fluid therapy (compound sodium lactate), maropitant (1 mg/kg IV q24h) and buprenorphine (0.02 mg/kg IV q6h). Overnight, the patient remained quiet, alert, and responsive, with a pain score of 2/10. Vital parameters, blood pressure and electrolytes remained within normal limits. Fluid balance was closely monitored, with intravenous fluid administration adjusted according to measured urine output, estimated ongoing losses, and hydration status. The urine remained grossly haematuric, and urine output reduced from 1.4 mL/kg/h post recovery to 0.9 mL/kg/h the following day. Forty-eight hours post recovery, the patient remained oliguric (urine output 0.8 mL/kg/h), with persistent azotaemia (creatinine 907 µmol/L [RI: 0–150]; urea 61.2 mmol/L [RI: 2.7–9.2]) prompting euthanasia according to the owner’s wishes.

**Figure 4:**
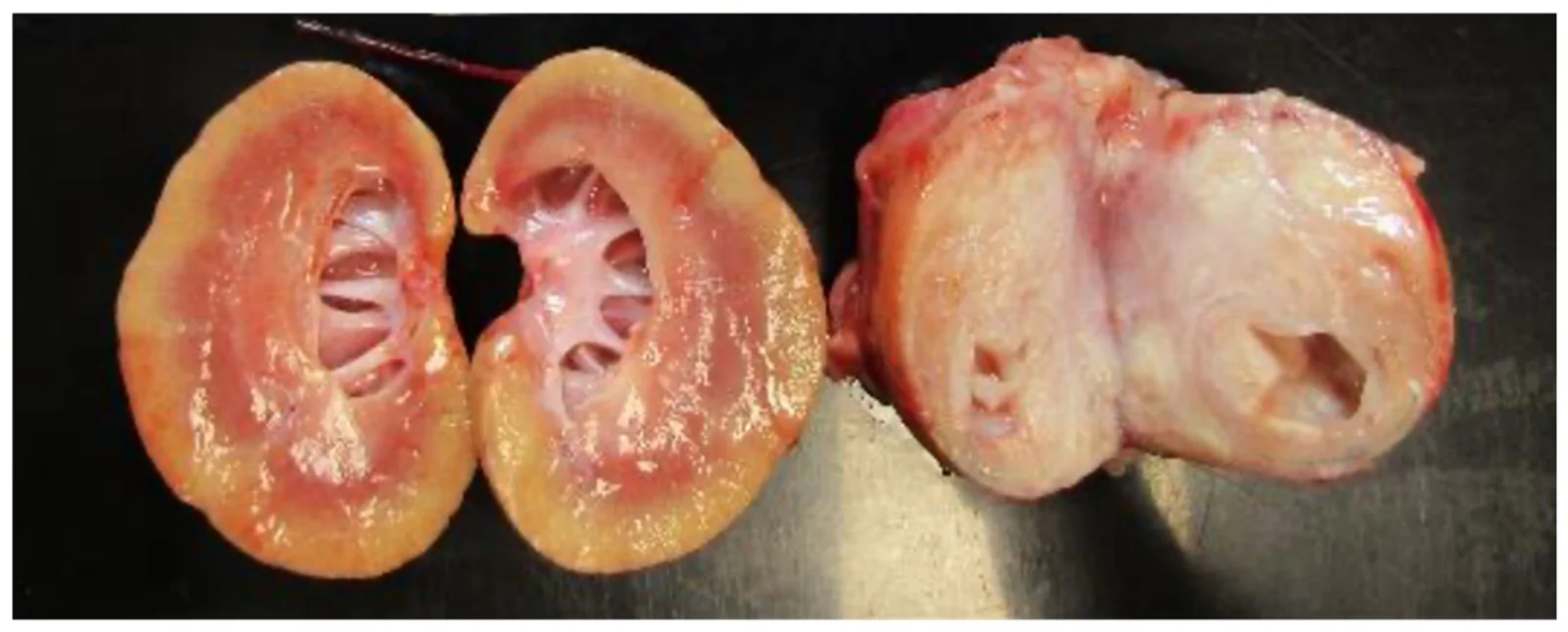
Post mortem image of left and right kidneys (left and right sides of image respectively) of 10-year-old Maine Coon with ectopic ureters and urothelial carcinoma. The right kidney is grossly abnormal, with obliteration of corticomedullary junction and multifocal discolorations on the cut surface, consistent with extensive neoplastic infiltration. In contrast, corticomedullary definition is maintained within the left kidney, which has multifocal to coalescing capsular depressions and a dark red capsule, consistent with chronic parenchymal disease rather than neoplasia.

A full post mortem examination was performed, in addition to an evaluation of the surgical biopsies, to systematically assess for primary disease extent and evidence of metastatic spread. The subcutaneous tissues, as well as respiratory, cardiovascular, alimentary, hematopoietic, reproductive, endocrine, musculoskeletal, and nervous systems were all examined grossly and histologically, with no evidence of neoplastic involvement; a redundant caudal vena cava was noted as an incidental vascular anomaly between the liver and the cranial pole of the right kidney. Multifocal omental nodules were noted, and the urinary system was confirmed to be grossly abnormal, with pathology affecting the kidneys, bladder, ureters and urethra. The right kidney was small and irregular and was adhered to the liver and right adrenal gland. The cut surface exhibited obliteration of the corticomedullary junction and multifocal discolouration, consistent with infiltrative disease (Figure 4). The right and left ureters were markedly and diffusely dilated, and surgical anastomosis at the bladder trigone was evident. The urinary bladder wall was thickened and exhibited multifocal to coalescing small haemorrhages. A full description of the gross post mortem features is given in Table 2.

**Figure 5:**
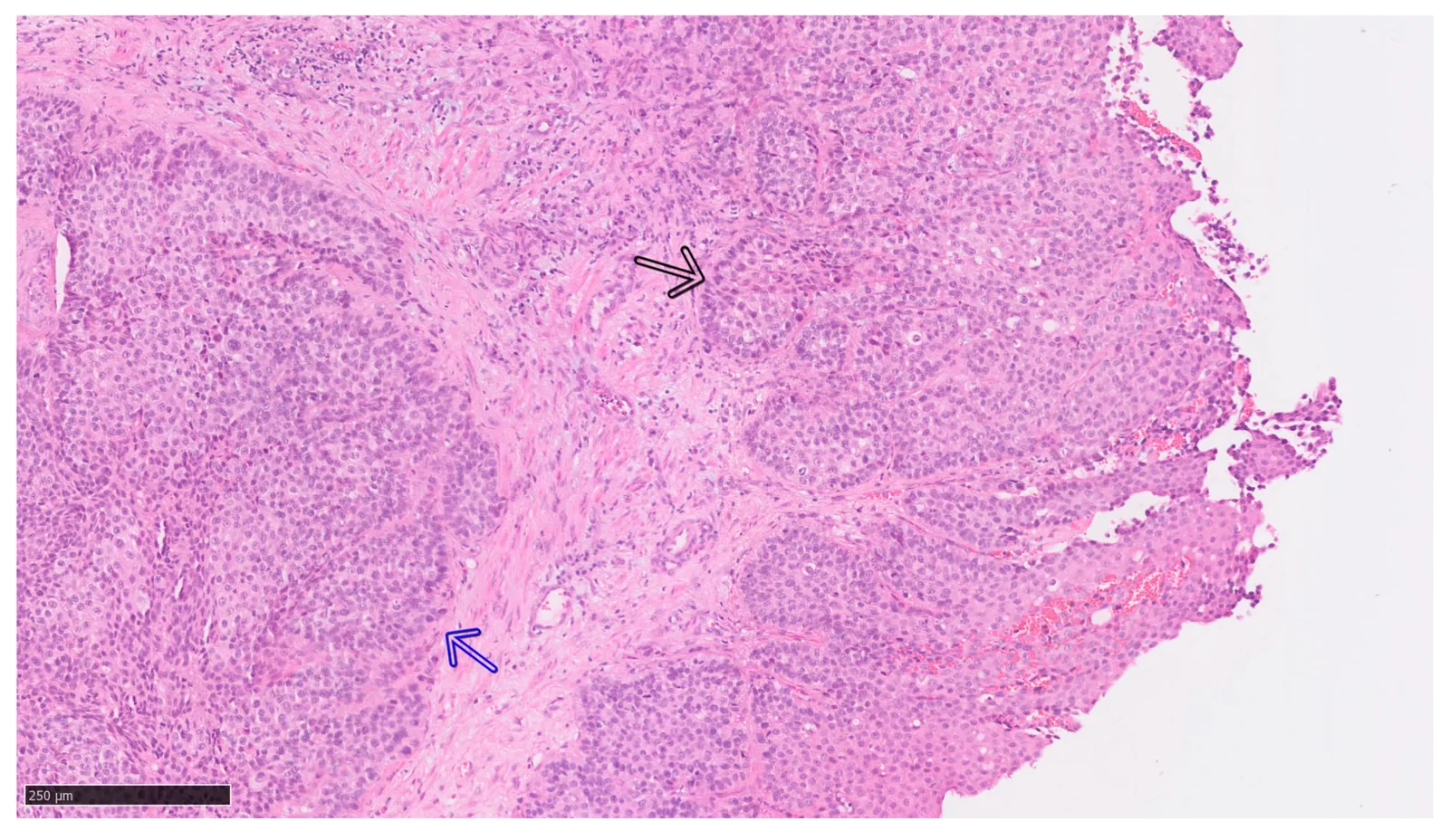
Urethral histopathology (H&E stain, 10× magnification) in a 10-year-old Maine Coon with ectopic ureters and urothelial carcinoma. A population of pleomorphic, polygonal neoplastic cells arranged in irregular trabeculae and nests, extensively replacing the mucosa (black arrow) and invading the submucosa (blue arrow).

**Figure 6:**
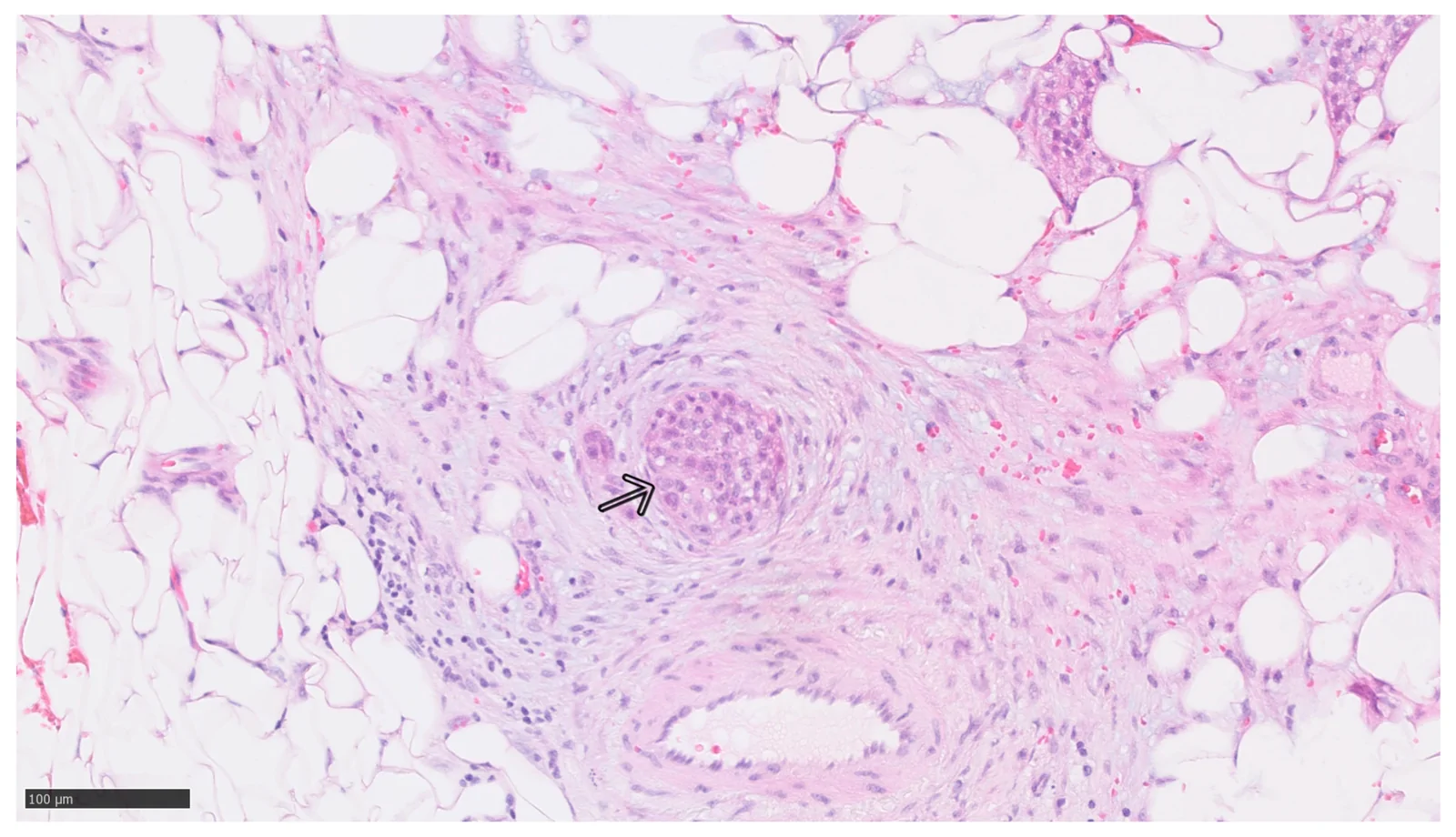
Omentum histopathology (H&E stain, 40× magnification) in a 10-year-old Maine Coon with ectopic ureters and urothelial carcinoma. Irregular nests of neoplastic cells (black arrow) multifocally infiltrate the omental biopsy, forming variably sized nodules with extensive coagulative to lytic necrosis and lymphatic invasion.

Histologically, an unencapsulated, poorly demarcated, densely cellular, infiltrative neoplasm compatible with carcinoma, composed of polygonal epithelial cells arranged in cords and trabeculae, was identified arising from the right ureteral and urethral urothelium at the level of the ectopic ureterovescical junction (Figure 5) and infiltrating the bladder wall. Similar urothelial cells with features of malignancy were noted in the right kidney and within the omental nodules (Figure 6). No histologic evidence of squamous or glandular differentiation was identified in the examined sections. The right kidney showed near-complete architectural effacement by coagulative necrosis and fibrosis, with only scattered neoplastic cells identified within this desmoplastic stroma. The left kidney showed multifocal moderate chronic lymphoplasmacytic interstitial nephritis with tubular necrosis, fibrosis, and intratubular calcium oxalate crystals, with no evidence of neoplastic infiltration. A full description of the histopathologic features is given in Table 2, the conclusions from which indicated a final diagnosis of urothelial carcinoma affecting the right ureter, bladder, urethra and right kidney with omental metastasis.

## 3. Discussion

Urothelial carcinoma (UC) is a rarely reported malignancy in cats, with ureteral involvement being particularly uncommon [2,4,28]. This case describes a previously unreported coexistence of UC with bilateral ectopic ureters, a congenital anomaly uncommonly reported in cats. The clinical presentation of progressive lower urinary tract signs is typical of both conditions, as is the noted development of hydroureters, hydronephrosis and urinary tract infection [2,13]. Whilst ectopic ureters are more common in female dogs, and certain dogs breeds are over-represented [29,30,31], no sex or breed predispositions have been established for ectopic ureters in cats. Nevertheless, multiple case reports have described ectopic ureters in the Maine Coon breed [32,33,34,35,36]. Similarly, unlike in dogs, where certain breeds are clearly over-represented for developing UC, case series reporting UC in cats describe a range of affected breeds [37].

Chronic inflammation has long been hypothesised to contribute to carcinogenesis [38], and, in humans, there is a well-documented association between chronic urinary pathology and the incidence of urinary tract neoplasia [9,10]. Moreover, evidence from the human literature supports a specific association between congenital anomalies of the kidney and urinary tract (CAKUT) and subsequent urinary tract malignancy. For example, one population-based cohort study following over 1.5 million recruits for up to 30 years found that a childhood diagnosis of CAKUT was associated with a significantly increased risk of developing renal, ureteral, and bladder malignancy in adulthood [39]. Notably, the proposed mechanism underlying this association extends beyond chronic urothelial inflammation, as the same genes governing normal nephrogenesis and ureteric bud maturation may, when disrupted, give rise to CAKUT. Thus, aberrant nephrogenesis has also been proposed to independently predispose the affected urothelium to tumorigenesis [39]. Although a specific genetic link has not been defined in patients with ectopic ureters, multiple human case studies have described urothelial carcinoma in patients with this congenital abnormality [17,18,21,22,40,41].

Veterinary evidence for these associations is sparce, consisting of a single case report describing an orthotopic ureterocele with concurrent urothelial carcinoma in a dog [6]. Whether a similar causal relationship exists in other veterinary species can therefore not be determined; however, the coexistence of UC and ectopic ureters in this patient raises this possibility. Alternatively, several non-mutually exclusive explanations should also be considered. The neoplasm may have arisen independently, with the two conditions representing coincidental rather than mechanistically linked pathology. Indeed, feline urothelial carcinoma has been reported to occur in the absence of any identifiable predisposing cause [37]. Whilst subclinical ectopic ureters can be associated with persistent urinary stasis and infection [13,17,18], it is notable that historical lower urinary tract signs such as incontinence, recurrent infection and urolithiasis were not reported, making their prior significance in this patient uncertain. Urinary stasis, urothelial disruption and compromised local defences, resulting from local tumour progression, could independently explain the presence of noted inflammation, infection, hydroureter and hydronephrosis; thus, anteriority is not confirmed [42,43]. Nevertheless, the hypothesis of a causal link raises the possible need for more proactive management and earlier intervention in animals with congenital urinary tract malformations.

Given the gross and histopathologic findings, it seems likely that the tumour arose in the vicinity of the ectopic ureterovesicular junction, with local extension into the adjacent right ureter, urethra and bladder, and with subsequent spread to the right kidney and omentum. Indeed, the distribution of neoplastic lesions likely reflects several recognised mechanisms of UC dissemination. Urothelial carcinoma is well-recognised for its propensity to disseminate via implantation metastasis, in which exfoliated neoplastic cells within the urine seed and implant along the urothelial surface at sites distant from the original tumour site; this mechanism plausibly explains the involvement of both the ectopic ureterovesicular junction region and right renal pelvis. In addition, histological identification of a tumour thromboembolus distinct from adjacent arterial and venous structures supports lymphatic invasion as a further route of local and potentially distant spread. This is consistent with the general tendency of carcinomas to metastasize preferentially via lymphatic rather than haematogenous pathways [44]. Finally, the presence of morphologically identical neoplastic cells within the omentum is consistent with transcoelomic implantation, a recognised route of peritoneal dissemination for carcinomas arising from or invading through a serosal surface [45,46]. The pattern of disease observed in this cat is therefore consistent with the combined effects of local infiltration, urinary implantation, lymphatic invasion, and peritoneal seeding and highlights the aggressive nature of feline UC. Indeed, as noted in this patient, the prognosis is generally poor, with reported median survival times in cats of 176 days with partially cystectomy and NSAID therapy or 46 days without treatment [2]. Whilst UC infiltration resulting in obstruction of the right ureter and urethra is assumed to have been the main driver of clinical deterioration in this cat, the presence of ongoing bacterial pyelonephritis cannot be excluded despite the negative urine cultures. Considering the cat’s history of chronic mild azotaemia and isosthenuria and the histopathologic changes evident in the contralateral (left) kidney, pre-existing chronic kidney disease and nephrolithiasis may have also played a role in the overall outcome.

This case report underscores the importance of considering the possibility of congenital urinary tract anomalies in older patients that have previously remained asymptomatic [17,18]. Whether the chronic sequalae of ureteral ectopia contributed to malignant transformation as has been proposed in the human literature remains speculative, but the importance of considering neoplasia as a possibility in patients with ectopic ureters experiencing progressive or non-responsive clinical signs is also highlighted.

## 4. Conclusions

This case report documents an unusual coexistence of ectopic ureters and UC in a cat, stressing the importance of including neoplasia in the differential diagnosis for patients with congenital urinary anomalies and progressive clinical signs. It highlights the aggressive nature of UC and the diagnostic and treatment challenges associated with late-stage disease. Given the body of evidence in human medicine, it also raises the possibility that congenital anatomic anomalies of the urinary tract in cats confer a risk of malignancy through a combination of altered urodynamics and a shared aberrant developmental pathway linking abnormal nephrogenesis to oncogenesis.

## Figures and Tables

**Table 1:** Urinalysis results of samples obtained by cystocentesis and bilateral pyelocentesis in 10-year-old Maine Coon with ectopic ureters and urothelial carcinoma at time of initial referral and at 4-week follow-up.

Sample Timing	Initial Referral (2 Weeks After 14-Day Course of Cephalexin)			5-Week Reassessment (1 Week After 4-Week Course of Marbofloxacin)		
Location	Left renal pelvis	Right renal pelvis	Bladder	Left renal pelvis	Right renal pelvis	Bladder
Urine SG	1.012	1.031	1.015	1.016	1.034	1.016
Dipstick evaluation	pH: 6 Leukocytes: - Protein: + Glucose: - Blood: - Hb: ++++ Ketones: - Bilirubin: -	pH: 9 Leukocytes: - Protein: +++ Glucose: - Blood: - Hb: ++++ Ketones: - Bilirubin: -	pH: 5 Leukocytes: - Protein: + Glucose: - Blood: - Hb: ++++ Ketones: - Bilirubin: -	pH: 6 Leukocytes: - Protein: - Glucose: - Blood: - Hb: + Ketones: - Bilirubin: -	pH: 7 Leukocytes: - Protein: + Glucose: - Blood: - Hb: +++ Ketones: - Bilirubin: -	pH: 5 Leukocytes: - Protein: + Glucose: - Blood: - Hb: +++ Ketones: - Bilirubin: -
Sediment examination	RBC: - WBC: >100/hpf Ep. cells: - Bacteria: + Crystals: -	RBC: >100/hpf WBC: >100/hpf Ep. cells: - Bacteria: ++ Crystals: -	RBC: 10/hpf WBC: >100/hpf Ep. cells: - Bacteria: ++ Crystals: -	RBCs: - WBC: >100/hpf Ep. cells: - Bacteria: ++ Crystals: -	RBC: 10/hpf WBC: >100/hpf Ep. cells: - Bacteria: ++ Crystals: -	RBCs: 10/hpf WBC: >100/hpf Ep. cells: ++ Bacteria: - Crystals: -
UPCR	0.86	5.91	0.5	0.16	0.67	0.50
Culture	*E. coli* > 2000 colonies	Negative	*E. coli* > 2000 colonies	Negative	Negative	Negative
Antibiotic susceptibility	Doxycycline Amoxicillin Enrofloxacin Marbofloxacin Cephalexin Amoxicillin-clavulanate TMPS Gentamicin	N/A	Doxycycline Amoxicillin Enrofloxacin Marbofloxacin Cephalexin Amoxicillin-clavulanate TMPS Gentamicin	N/A	N/A	N/A
Antibiotic resistance	Clindamycin	N/A	Clindamycin	N/A	N/A	N/A

SG = specific gravity, UPCR = urine protein creatinine ratio, RBC = red blood cell, WBC = white blood cell, Hb = haemoglobin, hpf = high powered field, Ep. cells = epithelial cells, UPCR = urine protein/creatinine ratio, TMPS = trimethoprim-sulfa, N/A = not applicable, + = positive, - = negative.

**Table 2:** Post mortem and surgical biopsy findings in a 10-year-old Maine Coon with ectopic ureters and metastatic urothelial carcinoma. Post- mortem examination was performed following bilateral ureteral reimplantation (ureteroneocystostomy), so that the gross findings reflect both the underlying disease processes and post-surgical changes. Findings above the black line represent histology following surgical intervention, whereas those below the black line represent post mortem findings.

Anatomical Site	Description of Gross Pathology	Histopathological Description	Histopathological Diagnosis
Bladder wall	Diffusely thickened on surgical palpation	There were mild mucosal erosions and ulceration with minimal multifocal hyperplasia. The submucosa was minimally infiltrated by perivascular lymphocytes and plasma cells, with few haemosiderin-laden macrophages. Moderate submucosal oedema and haemorrhage, mild-to-moderate multifocal congestion, and mild interstitial oedema of the tunica muscularis were present.	Chronic lymphoplasmacytic ulcerative cystitis with oedema
Urethra (junction of the right ectopic ureter and urethra)	Thickened, with abnormal tissue at the distal/ectopic insertion	Pleomorphic polygonal neoplastic cells arranged in irregular trabeculae and nests extensively replaced the mucosa and invaded the submucosa. No squamous or glandular differentiation was identified. Anisocytosis and anisokaryosis were moderate, with a markedly elevated mitotic count (34/2.37 mm^2^).	Urothelial carcinoma
Omentum	Multiple firm, white, discrete omental nodules	Irregular nests of pleomorphic polygonal neoplastic cells, similar to those in the urethral biopsy, multifocally infiltrated the omental biopsy, forming variably sized nodules with extensive coagulative to lytic necrosis and lymphatic invasion.	Urothelial carcinoma with lymphovascular invasion and fat necrosis
Right kidney	Orange, with obliteration of the cortex and medulla and corticomedullary distinction	Normal architecture was diffusely effaced by extensive coagulative necrosis and desmoplasia, with multifocal infiltration by polygonal epithelial neoplastic cells arranged in cords and trabeculae.	Marked necrosis with urothelial carcinoma
Left kidney	The cortex was yellow-green and the medulla pink, with good corticomedullary distinction	Multifocal fibrosis and lymphoplasmacytic infiltration replaced renal parenchyma, with tubular necrosis, degeneration or regeneration. Periglomerular fibrosis and occasional glomerulosclerosis were present. Multifocal tubular calcium oxalate crystals were identified, appearing as refractile, yellow sheaves/rosettes.	Chronic lymphoplasmacytic interstitial nephritis with tubular necrosis and intratubular calcium oxalate crystals
Right ureter	Pale pink appearance	Unencapsulated, densely cellular neoplasm arising from mildly hyperplastic urothelium and invading the lamina propria. Polygonal epithelial cells formed cords/trabeculae on fibrovascular stroma, with eosinophilic granular cytoplasm and vesiculate nuclei. Moderate anisocytosis/anisokaryosis; mitotic rate < 1/40× HPF. Occasional Melamed–Wolinska bodies were present.	Urothelial carcinoma
Bladder	Diffusely thickened, with coalescing red discoloration and haemorrhage.	The tunica muscularis was infiltrated by pleomorphic polygonal neoplastic cells arranged in irregular nests and sheets. No squamous differentiation was identified.	Urothelial carcinoma in the tunica muscularis of the bladder

## Data Availability

The data presented in this study are available on request from the corresponding author.
